# Meta-analysis of the accuracy of the serum procalcitonin diagnostic test for osteomyelitis in children

**DOI:** 10.1186/s12891-024-07716-3

**Published:** 2024-07-24

**Authors:** Han Qi, Dongsheng Zhu, Xiaodong Wang, Jian Wu

**Affiliations:** 1 Department of Emergency Surgery, The Second People’s Hospital of Lianyungang, Lianyungang, China; 2 Department of Pediatric Orthopedics, The First People’s Hospital of Lianyungang, Lianyungang, 222000 China; 3 Department of Orthopedics, Children’s Hospital of Soochow University, Suzhou, China; 4 Department of Pediatric, Xiangcheng District People’s Hospital, Suzhou, Jiangsu Province 215000 China

**Keywords:** Osteomyelitis, Procalcitonin, Diagnosis, Meta-analysis

## Abstract

**Objective:**

This study sought to assess the sensitivity, specificity, and predictive utility of serum procalcitonin (PCT) in the diagnosis of pediatric osteomyelitis.

**Methods:**

A systematic computer-based search was conducted for eligible literature focusing on PCT for the diagnosis of osteomyelitis in children. Records were manually screened according to the Preferred Reporting Items for Systematic Reviews and Meta-Analysis guidelines. Statistical analysis was performed using Review Manager software 5.3, Meta-disc software1.4, STATA 12.0, and R 3.4 software.

**Result:**

A total of 5 investigations were included. Of these, 148 children with osteomyelitis were tested for bacterial cultures in PCT. For PCT in the diagnosis of pediatric osteomyelitis, diagnostic meta-analysis revealed a pooled sensitivity and specificity of 0.58 (95% confidence interval (CI): 0.49 to 0.68) and 0.92 (95% CI: 0.90 to 0.93) respectively. The PCT had the greatest area under the curve (AUC) at 0.80 for the diagnosis of osteomyelitis in children. The Deeks’ regression test for asymmetry results indicated that there was no publication bias when evaluating publication bias (*P* = 0.90).

**Concusion:**

This study provided a comprehensive review of the literature on the use of PCT in pediatric osteomyelitis diagnosis. PCT may be used as a biomarker for osteomyelitis diagnosis; however, its sensitivity was low. It still needs to be validated by a large sample study.

## Introduction

Osteomyelitis in children and adolescents is an inflammation of bones induced by an acute bacterial infection [1]. Based on a study conducted by Agarwal et al. over the past decade, estimates of the total prevalence of bone and joint infections range from 2 to 13 per 10,000 youngsters [2]. However, it is often not clinically obvious where the infection-causing bacteria originated, making the colonization of the skin, mouth, or respiratory mucous membranes the most likely entry point for the infection, which is truly a diagnostic enigma in pediatric orthopedics [3]. Localized redness, swelling, suppuration, and other signs are the primary clinical indications of osteomyelitis [4]After an infection has taken hold, a rapid chain reaction of local and systemic inflammatory reactions occurs with the production of several inflammatory mediators. Laboratory serum inflammatory biomarkers had the features of accurate diagnosis in suspected instances of acute bone infections, both in pediatric and adult patients [5]. Procalcitonin (PCT) has been demonstrated in clinical studies to be a more accurate serum inflammatory biomarker for the detection of bacterial infections [6]. This biomarker exhibits the characteristics of a diagnostically reliable biomarker in suspected instances of acute bone infection. There were previously several findings in the literature that now suggest the usefulness of PCT in identifying acute osteomyelitis [7–9]. A research found PCT, at 0.4 ng/ml, was 85.2% sensitive and 87.3% specific in diagnosing septic arthritis and acute osteomyelitis; However, in comparison, PCT at conventional cut-off of 0.5 ng/ml is 66.7% sensitive and 91% specific, it seemed 0.4 ng/ml more suitable for diagnosis osteomyelitis [7]. In pyogenic spondylodiscitis, Italian scholars have found that PCT also has certain diagnostic value, especially when PCT was greater than 0.11ng/ml, it indicated poor prognosis [10]. Given the increasing use of PCT in the diagnosis of pediatric osteomyelitis, we conducted a thorough and quantitative review of current relevant literature reports to provide a scientific basis for the clinical use of PCT in the diagnosis of pediatric osteomyelitis.

## Materials and methods

### Search strategy

PubMed, Embase and the Cochrane Library were used to identify articles eligible for the meta-analysis (last search: April 01, 2023), with the following subject terms: “osteomyelitis” or “bone infection” and “procalcitonin” or “PCT” and “adolescent” or “children” or “pediatrics”. We also reviewed the reference lists of previous systematic reviews to identify additional studies that might have been eligible for inclusion in the analysis. Reference lists of relevant reviews and previous meta-analyses were also searched.

### Inclusion and exclusion criteria

The analysis comprised the following studies: (a) objective was to assess PCT’s diagnostic utility for osteomyelitis in children; (b) studies had a sufficient amount of data to build two-by-two contingency tables; (c)age under 18;(d) gold standard for the diagnosis of osteomyelitis was pathogen isolation or culture. The following studies were excluded from the analysis: review articles, case reports, clinical guideline and animal experiments.

### Quality assessment

Quality Assessment of Diagnostic Accuracy Studies-2 (QUADAS-2) tool was used to assess the methodological quality of the included studies.

### Literature screening and data extraction

Two reviewers reached an agreement on which studies should ultimately be included in the analysis after each of them separately assessed the studies that were eligible for inclusion. Negotiations or the assistance of a third researcher were used to resolve disagreements. Most of the retrieved information consisted of the following: study, year, country, age and sample, PCT cut-off and true positive (TP), false positive (FP), true negative (TN), and false negative (FN) of PCT.

### Criteria of heterogeneity test

Threshold and non-threshold effects were the primary sources of variability in diagnostic tests. On the receiver operating characteristic (ROC) plane graph, sensitivity and specificity displayed a “shoulder-arm” point distribution when the threshold effect was present. Otherwise, there was no correlation. When there was no threshold effect, individual evaluation metrics, such as sensitivity and specificity, can be combined immediately. When threshold effect was present, Summary ROC (SROC) curve approach should be used to calculate the area and Q-index.

### Statistical methods

Sensitivity, specificity, positive likelihood ratio (LR), negative likelihood ratio (LR), and diagnostic odds ratio (DOR) were calculated. Spearman correlation analysis was used to detect the threshold effect, and the SROC curve approximation was used to calculate the area and Q-index if threshold effect was present. I^2^ values were used to measure the heterogeneity between studies. I^2^ values > 50% suggested that there was significant heterogeneity present. We used the Deeks’ regression test for funnel plot asymmetry to examine for potential publication bias. Statistical analysis was performed using Review Manager Software 5.3, Meta-disc software1.4, STATA 12.0, and R 3.4 software.

## Results

### Basic characteristic

**Fig. 1:**
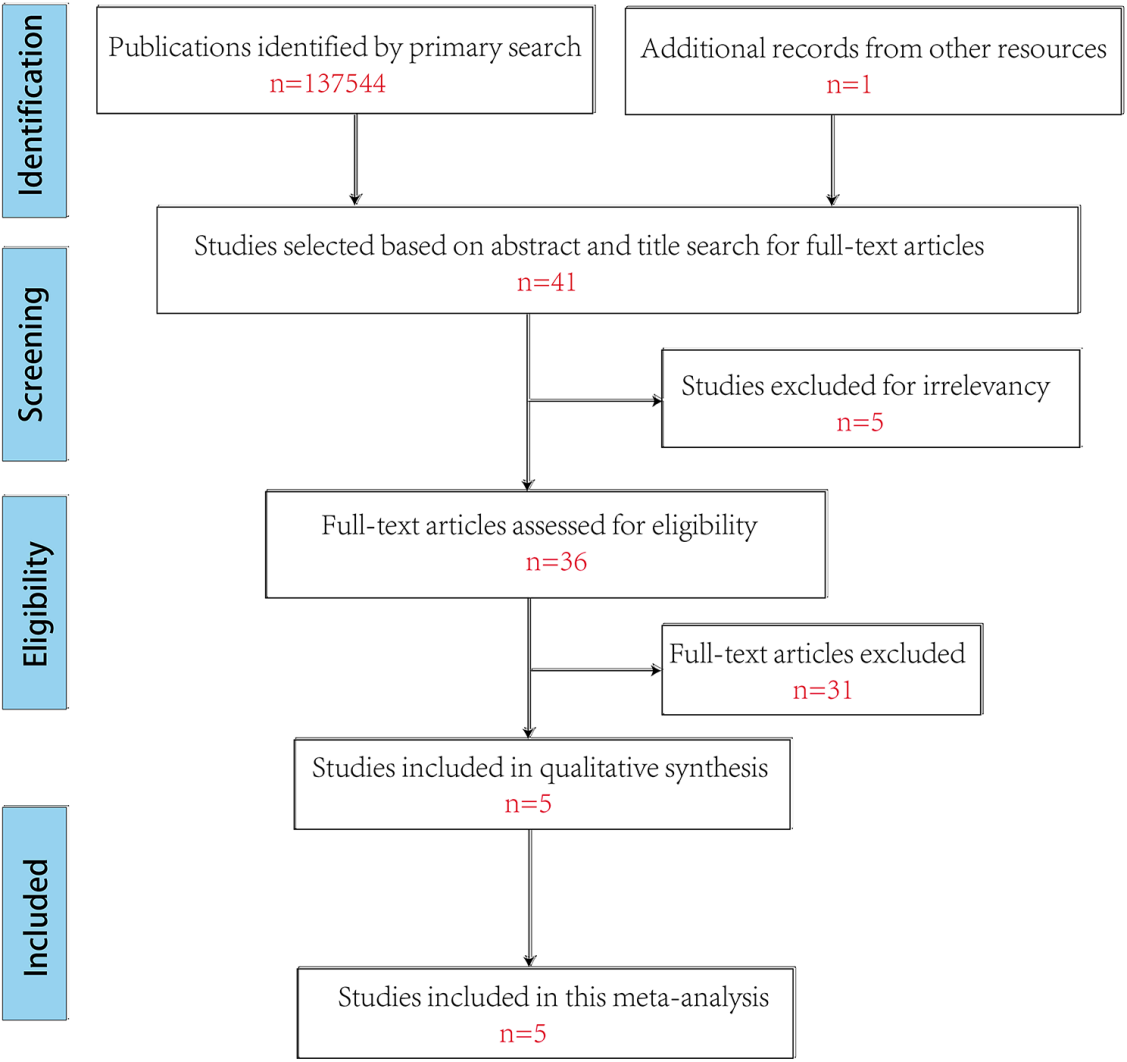
Diagram of workflow in the systematic review and meta-analysis

The above searches yielded a total of 137,544 documents; however, only 5 articles met the inclusion requirements (Fig. 1) [11–15]. Finally, 5 articles came from USA, France, Israel, China and South Africa included 1235 children between 2005 and 2022 (Tables 1 and 2).

**Table 1:** Basic characteristic of the included studies

Author	Year	Nation	Age(year)	Study Design	Pro-calcitonin detection method	Instrument company
Butbul-Aviel	2005	Israel.	7.9 (7.6 ± 5.5)	prospective	A semi-quantitative rapid immunoassay	BRAHMS
Faesch	2009	France	4(0.1–14)	retrospective	An automatic quantitative method	BRAHMS
Greeff	2013	South Africa	< 14	prospective	Pro-calcitonin sensitive kit	BRAHMS
Cui	2017	China	6.50 ± 3.44	prospective	Micro-particle enzyme immunoassay	BRAHMS
Lyons	2022	USA	7(4–11)	prospective	Sensitive compact immunoassay	BRAHMS

**Table 2:** Basic data of the included studies

Author	Year	Sample	osteomyelitis	True positive	False positive	False negative	True negative	Sensitivity	Specificity	Cut-off
Butbul-Aviel	2005	44	12	7	3	5	29	58.33%	90.62%	0.5ng/mL
Faesch	2009	339	20	5	10	15	309	25.00%	96.9%	0.5ng/mL
Greeff	2013	33	4	3	11	1	18	75.00%	62.07%	0.5ng/mL
Cui	2017	172	92	44	35	13	80	77.17%	69.47%	0.356 ng/mL
Lyons	2022	647	20	7	32	13	595	35.00%	96.86%	0.5ng/mL

### Quality evaluation

**Fig. 2:**
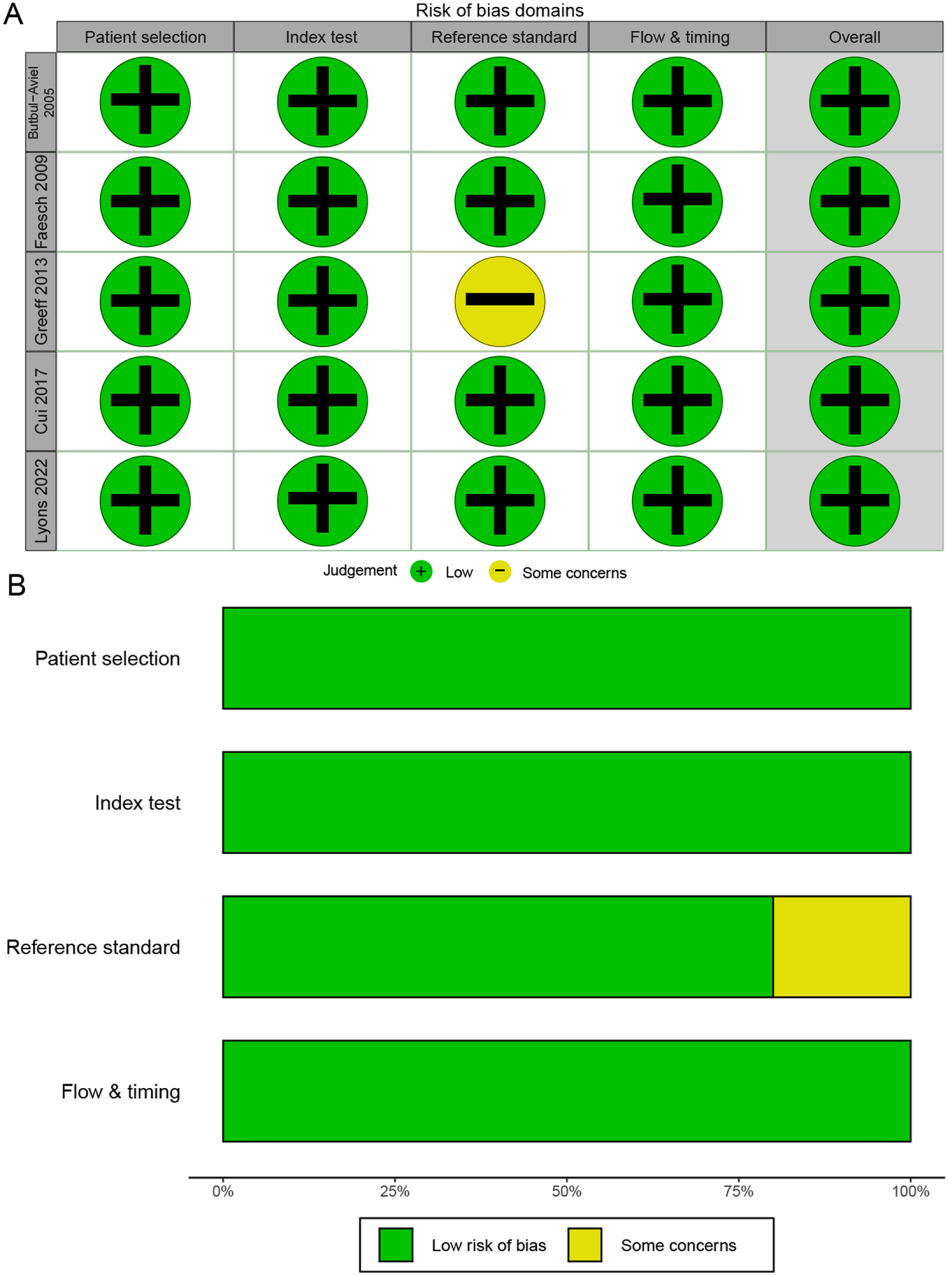
Methodological quality summaries

Evaluation of literature quality based on QUADAS-2 entries, including patient selection, index test, reference standard, and flow & timing, only one study was described as ambiguous in the reference criteria (Fig. 2).

### Threshold effect detection

**Fig. 3:**
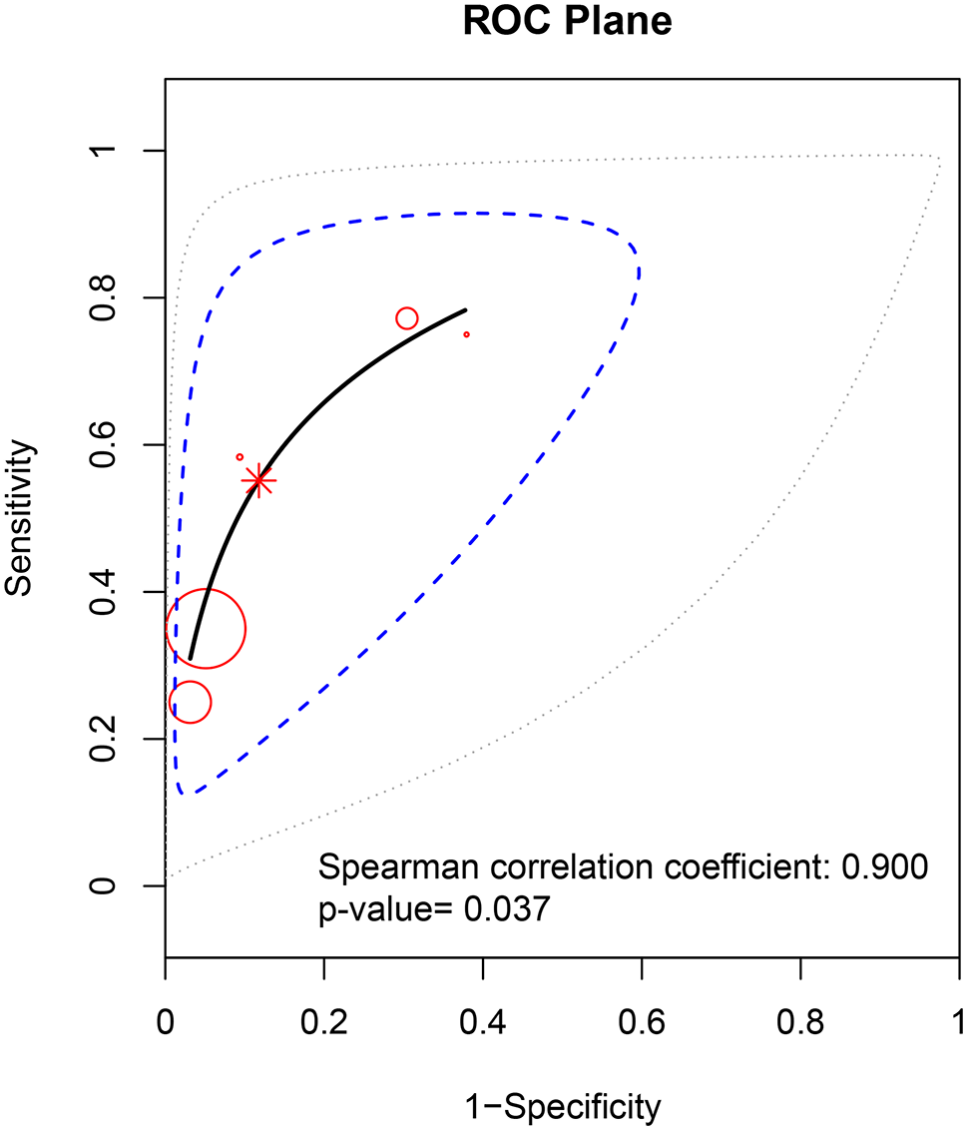
ROC plane graph for threshold effect detection

This can lead to a threshold effect due to the different critical values of the PCT. Therefore, the first step is to test whether there is a threshold effect in this diagnostic experiment. The Spearman correlation coefficient was 0.9(*P* = 0.037) and ROC plane graph displayed a “shoulder-arm” point distribution, indicating a threshold effect in PCT detection of pediatric osteomyelitis (Fig. 3). Therefore, the SROC curve approximation should be employed to calculate the area and Q-index.

### The value of PCT in diagnosing osteomyelitis in children

**Fig. 4:**
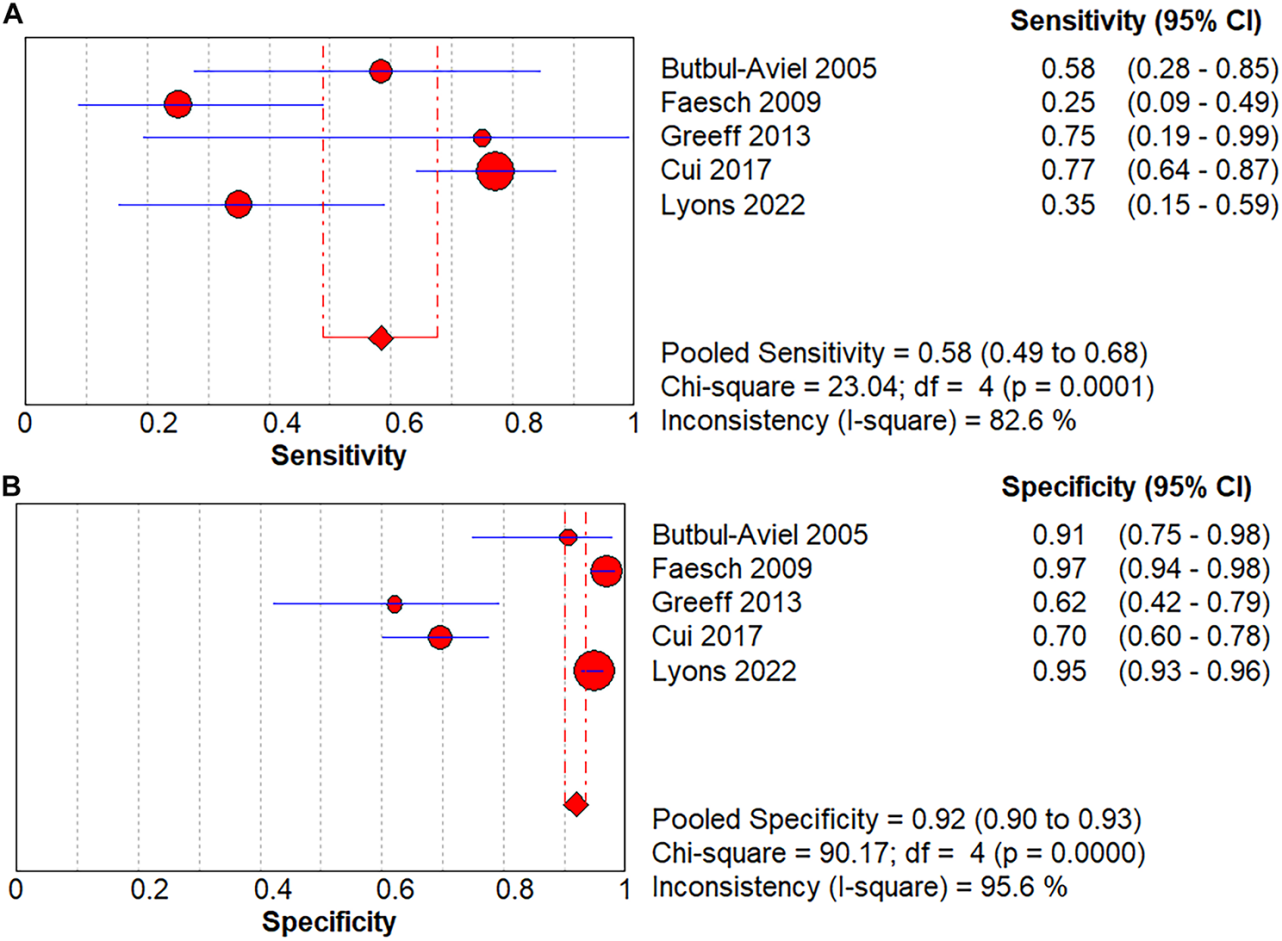
(**A**) Forest of sensitivity for procalcitonin diagnosis of children with osteomyelitis; (**B**) Forest of specificity for procalcitonin diagnosis of children with osteomyelitis

**Fig. 5:**
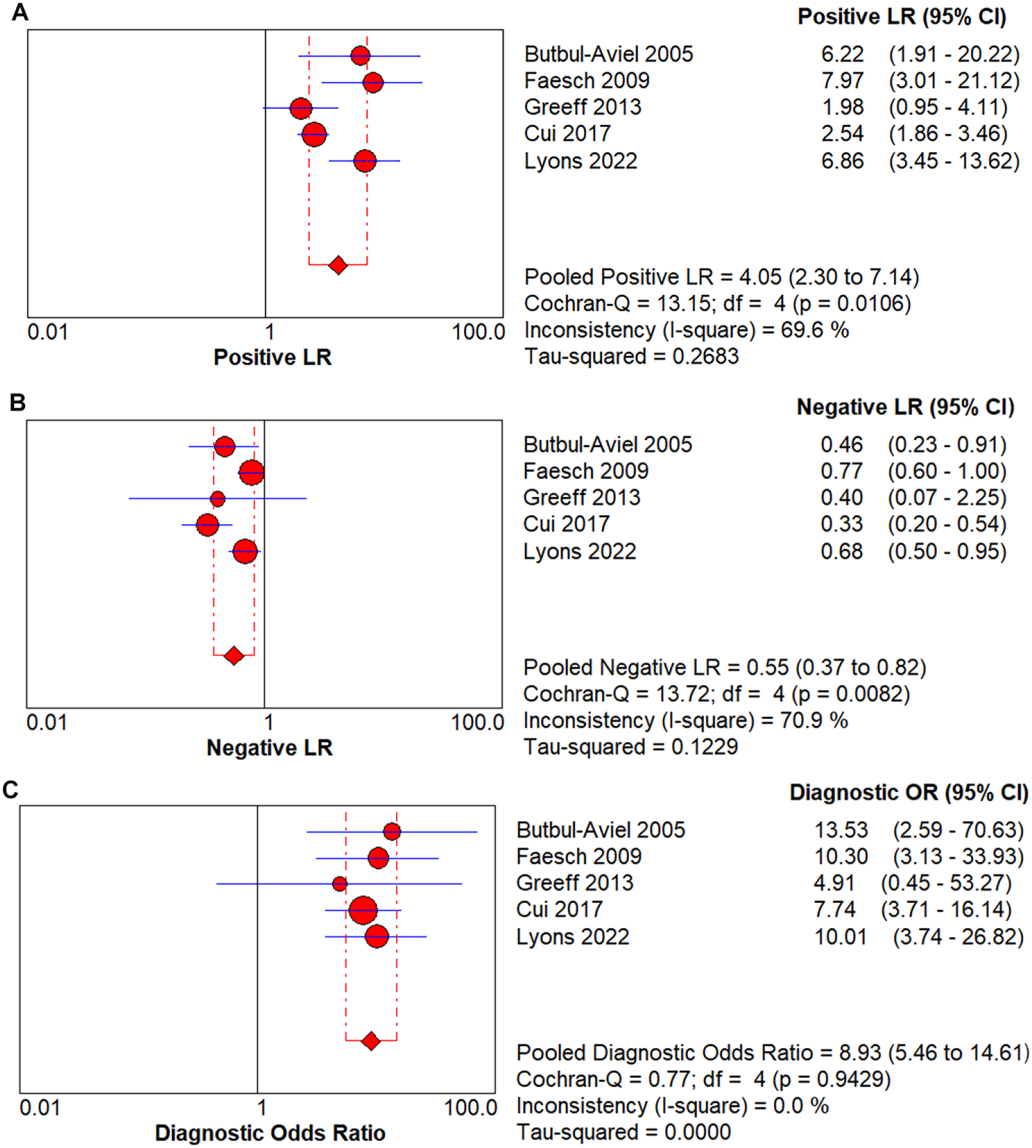
(**A**) Forest of positive likelihood ratio for procalcitonin diagnosis of children with osteomyelitis; (**B**) Forest of negative likelihood ratio for procalcitonin diagnosis of children with osteomyelitis; (**C**) Forest of diagnostic odds ratio for procalcitonin diagnosis of children with osteomyelitis

**Fig. 6:**
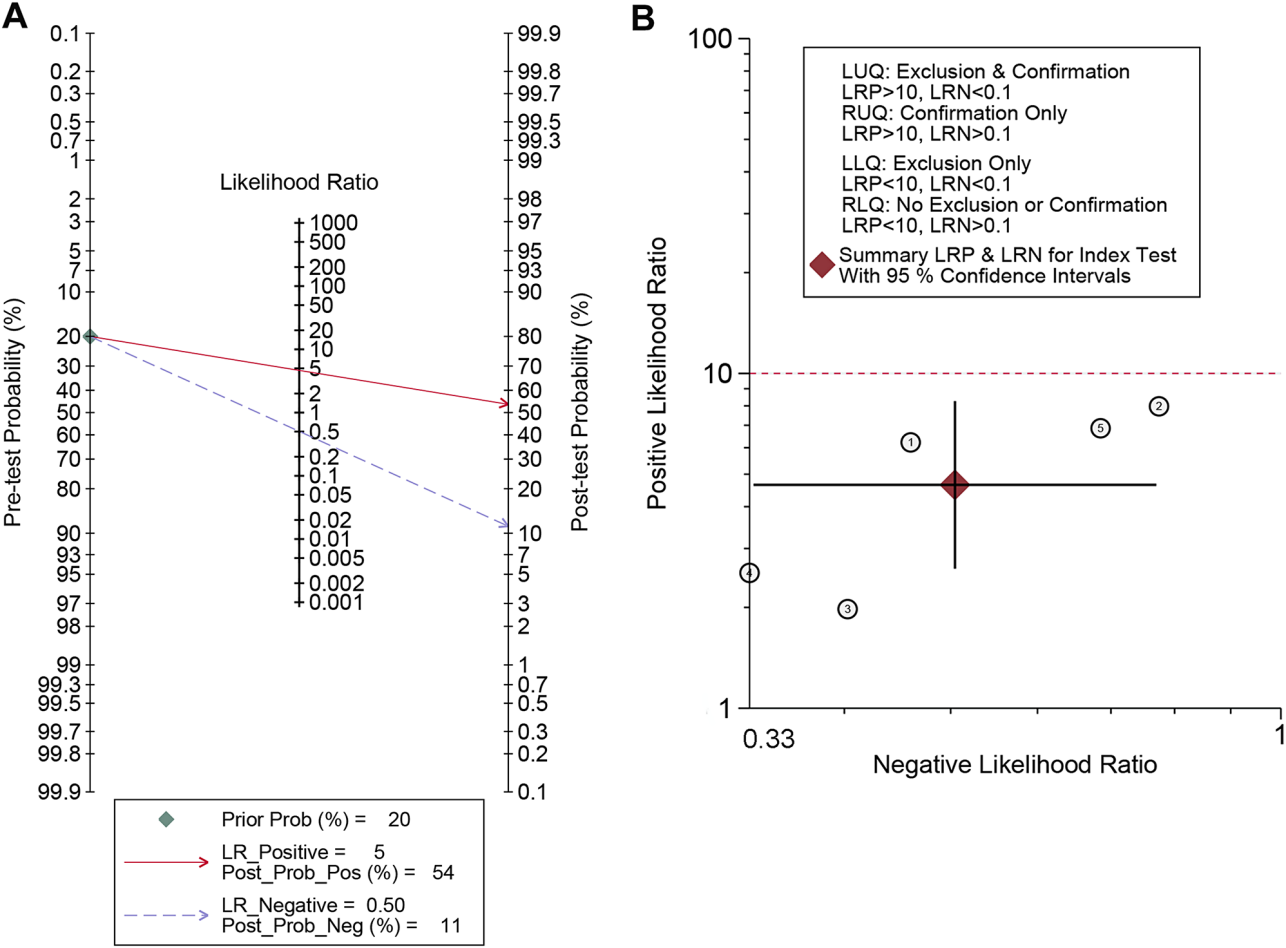
(**A**) Fagan nomogram of PCT for diagnosis of children with osteomyelitis; (**B**) The value gram of positive likelihood ratio and negative likelihood ratio for procalcitonin diagnosis of children with osteomyelitis

**Fig. 7:**
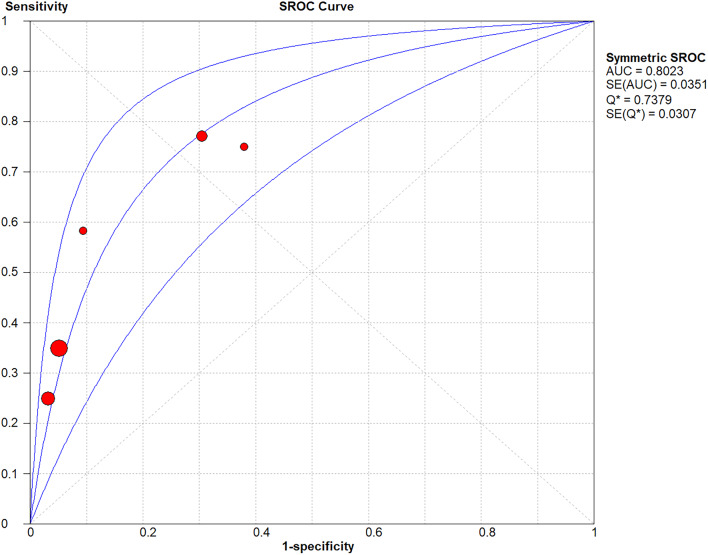
Summary receiver operating characteristics curve for procalcitonin diagnosis of children with osteomyelitis

In all 5 trials included in the final meta-analysis, PCT had a pooled sensitivity of 0.58 (95% Confidence interval (CI), 0.49–0.68) to osteomyelitis diagnosis in children (Fig. 4A) and a pooled specificity of 0.92 (95% CI, 0.90–0.93)to osteomyelitis diagnosis in children (Fig. 4B).The pooled positive LR was 4.05(95% CI, 2.30–7.14)(Fig. 5A), the pooled negative LR was 0.55 (95% CI, 0.37–0.82)(Fig. 5B), and the diagnostic OR was 8.93, 95% CI (5.46 − 14.61)(Fig. 5C), for the detection of osteomyelitis by PCT. Fagan’s nomogram showed the change in the predictive power of the PCT in pediatric osteomyelitis diagnosis after meta-analysis (Fig. 6A)and its predictive utility (Fig. 6B).Based on the SROC curve of the PCT, the AUC of the PCT was found to be 0.80 (Fig. 7).

### Publication bias

**Fig. 8:**
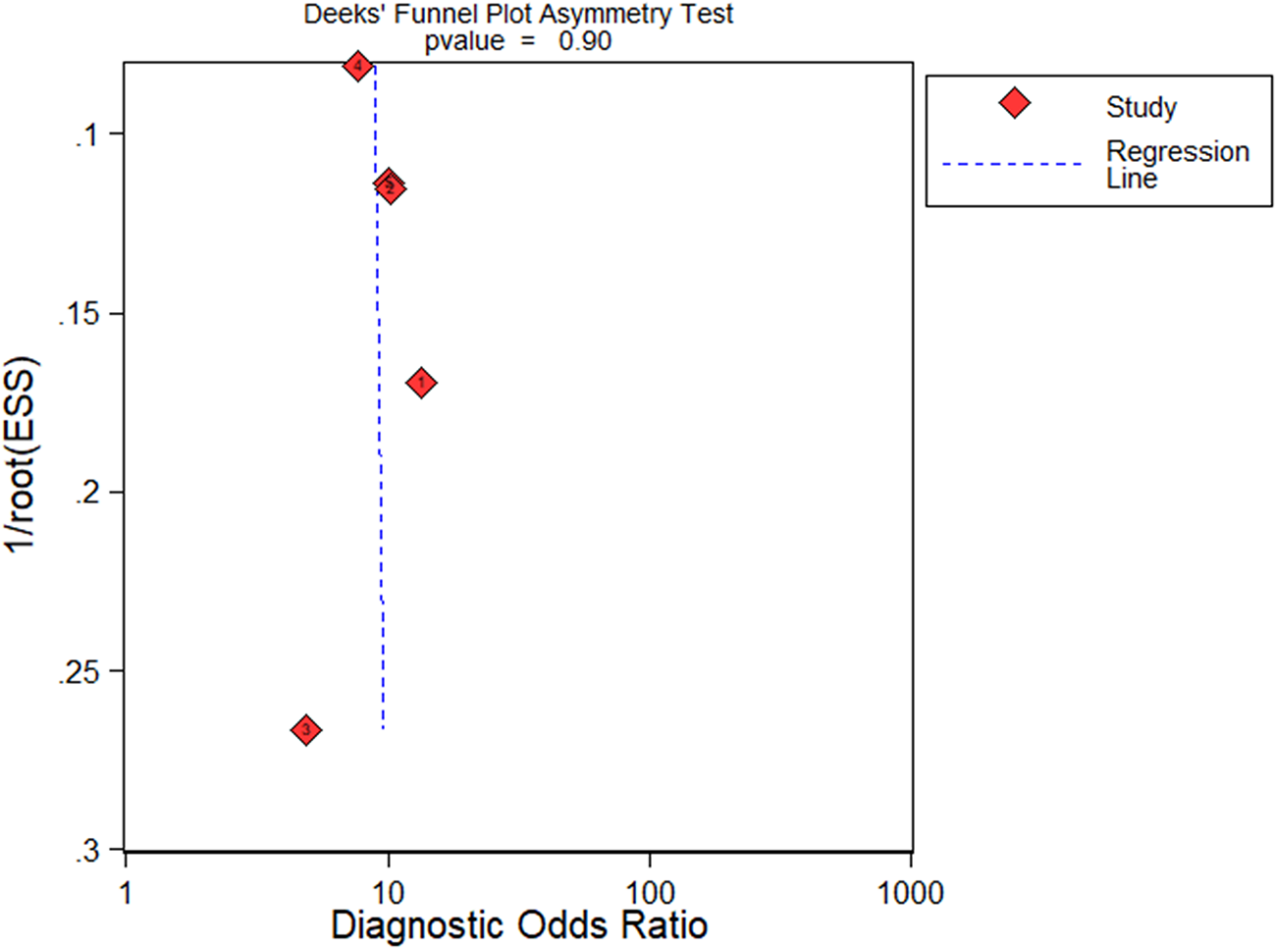
Deeks’ funnel plot for procalcitonin diagnosis of children with osteomyelitis

Using Deeks’ regression test for asymmetry to detect publication bias (*P* = 0.9,> 0.05), we found that there was no discernible publication bias in the papers that were part of the study (Fig. 8).

## Discussion

Osteomyelitis is most common in the long bones of the limbs, such as the femur, tibia, humerus, and radius, especially the femur and tibia [16]. It is a bacterial infection that spreads into bone tissue via the bloodstream, trauma, local diffusion, or surgery [17]. Microbiological examinations of children were previously used to determine the diagnostic criteria for osteomyelitis. However, bacterial culture typically takes several days, which is not conducive to the early diagnosis of acute osteomyelitis in children. Thus, serum inflammation biomarkers play a potential role in clinical practice for patients with suspected osteomyelitis, of which PCT is one [6]. Osteomyelitis must be diagnosed and treated at an early stage, especially for children and adolescents who are still growing and developing [18]. If it develops into chronic osteomyelitis, it can potentially impair a child’s bone growth and can remain morbid, sometimes taking years to heal [19]. 

PCT is a precursor peptide of calcitonin that is stable in humans [20]. Within a few hours of contracting a bacterial infection, children emit huge amounts of interleukins and tumor necrosis factor, which will boost the expression of PCT gene [21]. Since PCT is a sensitive biomarker of an important inflammatory component in bacterial infections, it is now routinely employed as a marker of infection [22]. Following an extensive literature review, we did not find any meta-analysis of PCT in pediatric osteomyelitis diagnosis, only a similar report [23]. However, this paper incorporated pediatric osteomyelitis and purulent arthritis into one set of data, which inevitably leads to a bias. To investigate the usefulness of PCT as a diagnostic biomarker for osteomyelitis in children, we performed a meta-analysis that eventually included 5 studies. We used pooled data from 5 studies enrolling a total of 148 children with osteomyelitis presentation. This study contained one retrospective study and four prospective trials of excellent overall quality. Our meta-analysis indicated a pooled sensitivity of 58% and specificity of 91% for osteomyelitis diagnosis in children. The findings suggested that the rate of misdiagnosis of osteomyelitis in children is minimal, but the rate of missed diagnosis is high.

Pooled likelihood ratio estimation: positive LR and negative LR were used to calculate the post-test probabilities to make our results more clinically informative. They are relatively independent and clinically significant evaluation indicator of the efficacy of diagnostic trials [24]. The meta-analysis indicated a pooled positive LR was 4.05, and a pooled negative LR was 0.55. In addition, Fagn’s nomogram showed that given a pre-test probability of 20%, a pooled negative LR of 0.50 reduces the post-test probability to 11%. Thus, only 1 out 9 patients with negative PCT results may end up with osteomyelitis. However, as reported in previous literature, when the pooled positive LR > 10, and the pooled negative LR < 0.1, the likelihood of diagnosing or ruling out a certain disease is significantly increased [25]. This suggests that the PCT still has some shortcomings in its application to the diagnosis of pediatric osteomyelitis. In 2021, a meta-analysis examined the value of C-reactive protein (CRP) in the diagnosis of bone and joint infections in children and adolescents. They found a sensitivity and specificity of 0.86 and 0.90 for CRP in the diagnosis of bone and joint infections in children and adolescents, respectively; however the positive LR and negative LR were 5.3 and 0.1, respectively [26]. This indicates that the ability to diagnose bone and joint infections in children and adolescents using CRP alone remains inadequate. ​Similar shortcomings were found in our meta-analysis of PCT, so a combined test is still necessary. ​In addition, there is still no consensus on the best PCT cut-off for diagnosing inflammatory bone and joint disease. PCT at the conventional cut-off of 0.5 ng/ml; however, some scholars have suggested that 0.4 ng/ml is more appropriate for the diagnosis of osteomyelitis [7]. In pyogenic spondylodiscitis, Italian scholars have found that the PCT also has some diagnostic value, especially when the PCT is greater than 0.11ng/ml, indicating a poor prognosis [10]. Therefor, more high-quality studies are necessary in the future to further investigate the role of PCT in the diagnosis of pediatric osteomyelitis. When the AUC is lower than 0.5, it indicates no diagnostic value; 0.5 ~ 0.7 indicates a low diagnostic value; 0.7–0.9 indicates a high diagnostic value; When it is higher than 0.9, the diagnostic accuracy is highest [27]. The results of this study showed that the PCT AUC reaches 0.80, indicating high diagnostic accuracy. The Q-index is the value corresponding to the point closest to the upper left corner of the SROC curve, where sensitivity and specificity are equal. The higher the -Q-index, the higher the accuracy of the diagnostic test. The Q-index of this study was 0.737, which, combined with the AUC, indicats that PTC is of good diagnostic value for the diagnosis of osteomyelitis in children. Our study provides a comprehensive review of the literature on the application of PCT in the diagnosis of pediatric osteomyelitis and yields several important conclusions. PCT emerges as a potential biomarker for osteomyelitis diagnosis, with its elevated levels showing a certain degree of correlation with the occurrence of the disease. This offers a novel diagnostic approach. However, given the low diagnostic sensitivity of the PCT, it is still best to combine other indicators for the detection of pediatric osteomyelitis. Despite the limited sensitivity of PCT, our findings still make significant contributions to the current understanding of PCT in pediatric osteomyelitis diagnosis. Firstly, our review provides clinicians with comprehensive information on the application of PCT in pediatric osteomyelitis diagnosis, helping them better understand the advantages and limitations of this biomarker. Secondly, our findings highlight the direction for future research, which is to further validate the value of PCT in pediatric osteomyelitis diagnosis through large-sample studies and explore possible methods to improve its sensitivity.

The significant findings of our study have important implications for the clinical management of pediatric osteomyelitis. Our results indicate that PCT may serve as a reliable biomarker for the diagnosis of this condition, with potential to improve diagnostic accuracy and reduce unnecessary antibiotic exposure. This has significant value given the risks associated with misdiagnosis and overtreatment of pediatric osteomyelitis. Moreover, our work builds upon and extends the current understanding of PCT as a diagnostic marker in pediatric osteomyelitis. While previous studies have examined the utility of PCT in this context, our study provides additional insights by focusing specifically on pediatric patients and analyzing a larger sample size. This allows us to more precisely characterize the diagnostic performance of PCT in this patient population.

However, it is important to acknowledge that our study has limitations, including the retrospective nature of the analysis and the potential for confounding factors. Limitations of the study: (1) The design of the included studies contained prospective research and perspective studies, which may have had some impact on the results; (2) The threshold values for inclusion in the study were different, and different thresholds may still affect the sensitivity and specificity of the PCT diagnosis; 3)Spearman correlation coefficients suggested the presence of threshold effects, and subgroup analyses were not performed due to the limited literature included; 4)This study included only English-language literatures, and may have been language bias. Future prospective studies with larger sample sizes and rigorous methodological designs will be necessary to further validate our findings and determine the optimal use of PCT in pediatric osteomyelitis diagnosis. Additionally, we can consider incorporating more clinical parameters and biomarkers, such as white blood cell count, CRP, and erythrocyte sedimentation rate, to enhance the accuracy and reliability of diagnosis. Futhermore, we can explore new detection techniques, such as gene- or proteome-based detection methods, to potentially improve the sensitivity of PCT in pediatric osteomyelitis diagnosis.

## Conclusion

This study provided a comprehensive assessment of the existing literature on the diagnosis of osteomyelitis in children using PCT, which may serve as a biomarker for osteomyelitis diagnosis. However, the PCT has a low sensitivity to pediatric osteomyelitis diagnosis. Hence, its use as a biomarker for pediatric osteomyelitis detection alone is not recommended and needs to be combined with other detection methods. Due to the limitations in the number and quality of included studies, a large number of high-quality studies are required in the future to further explore the application value of their combined diagnostics.

## Data Availability

Datasets used and/or analysed during the current study are available from the corresponding authors upon reasonable request.

## Abbreviations

PCT: Procalcitonin
QUADAS-2: Quality Assessment of Diagnostic Accuracy Studies-2
TP: True positive
FP: False positive
TN: True negative
FN: False negative
ROC: Receiver operating characteristic
SROC: Summery receiver operating characteristic
LR: Likelihood ratio
DOR: Diagnostic odds ratio
CI: Confidence interval
